# Recent advances in the understanding of trimeric autotransporter adhesins

**DOI:** 10.1007/s00430-019-00652-3

**Published:** 2019-12-21

**Authors:** Andreas R. Kiessling, Anchal Malik, Adrian Goldman

**Affiliations:** 1 Astbury Centre for Structural Molecular Biology, School of Biomedical Science, University of Leeds, Leeds, LS2 9JT England, UK; 2 Faculty of Biological and Environmental Sciences, University of Helsinki, FIN-0014 Helsinki, Finland

**Keywords:** Trimeric autotransporter adhesins (TAAs), Autotransporter, Protein secretion, Adhesins, *Burkholderia pseudomallei*, BpaC, Protein translocation, Protein export, BAM, Gram-negative bacteria, Autotransport, Outer membrane, Transmembrane β-barrel, Type V secretion, Bacterial proteins, Bacterial outer membrane proteins, Protein folding, Protein structure, Protein transport, Virulence factors/chemistry

## Abstract

Adhesion is the initial step in the infection process of gram-negative bacteria. It is usually followed by the formation of biofilms that serve as a hub for further spread of the infection. Type V secretion systems engage in this process by binding to components of the extracellular matrix, which is the first step in the infection process. At the same time they provide protection from the immune system by either binding components of the innate immune system or by establishing a physical layer against aggressors. Trimeric autotransporter adhesins (TAAs) are of particular interest in this family of proteins as they possess a unique structural composition which arises from constraints during translocation. The sequence of individual domains can vary dramatically while the overall structure can be very similar to one another. This patchwork approach allows researchers to draw conclusions of the underlying function of a specific domain in a structure-based approach which underscores the importance of solving structures of yet uncharacterized TAAs and their individual domains to estimate the full extent of functions of the protein a priori. Here, we describe recent advances in understanding the translocation process of TAAs and give an overview of structural motifs that are unique to this class of proteins. The role of BpaC in the infection process of *Burkholderia pseudomallei* is highlighted as an exceptional example of a TAA being at the centre of infection initiation.

## Introduction

The ongoing antibiotic crisis affects all aspects of our life: the chief medical officer of the UK, Dame Sally Davies, says the threat is as great as that from climate change [1], so developing new therapies and strategies to combat “superbugs” is both essential and urgent. For bacterial pathogens, adhesion and aggregation are the first steps of evasion [2]. In Gram-negative bacteria, Type V secretion systems (Va–Ve) play an important role in these initial steps of host infiltration [3]. Proteins of the type Vc secretion system, also known as trimeric autotransporters (TAAs), are involved in several aspects of the infection process of gram-negative bacteria, not only in adhesion and biofilm formation, but also in immune evasion. In other words, TAAs serve as one of the important factors of pathogenesis, so the study of their biogenesis, architecture and adhesion mechanism is of great importance.

The first autotransporter (Type Vb IgA protease from *Neisseria gonorrhoeae*) was described in 1987 [4], and they were so named because it was thought that the information for their secretion and translocation was purely stored in the protein sequence itself and did not depend on any external factors. This idea has been challenged over the last 20 years by the evidence of autotransporter interaction with periplasmic chaperones like DnaK [5], the involvement of the translocation and assembly module (TAM) in the correct folding of the autotransporter Antigen 43 (Ag43) [6], and the final outer membrane insertion being driven by the β-barrel assembly machinery (BAM) [7].

In this review, we briefly describe the domain architecture of trimeric autotransporter adhesins (TAAs), highlight the importance of biogenesis on their final assembly in the outer membrane of gram-negative bacteria and discuss the contribution of TAAs in the infection process, using as an example the involvement of the trimeric autotransporter BpaC from *Burkholderia pseudomallei* in the attachment and proliferation of this gram-negative bacterium.

### Translocation pathway of trimeric autotransporters

The process of type V secretion can be separated into three steps. The Sec machinery first enables transport of the protein through the cytoplasmic membrane. The autotransporter is then passed to various periplasmic chaperones that keep it in an export-competent state until finally it is recognized by the BAM complex and inserted into the outer membrane of gram-negative bacteria [8].

Although much progress has been made in recent years in unravelling each individual stage of the translocation process of autotransporters, a unified model that explains all experimental data collected is still missing. It is highly likely that all autotransporters share a common translocation mechanism for the following reasons: first, they share features across all the different stages of translocation. This has allowed one to connect individual discoveries from one subset of the family to others. Second, they share an N-terminal signal sequence for Sec-dependent translocation into the periplasm, a central passenger domain with various functional domains, and a conserved outer membrane channel-forming β-barrel at the C-terminus that is required for the export of the passenger domain to the outside [9]. Third, the C-terminal domain always resembles OMP85 family of proteins, which are monomeric proteins with a structurally conserved 12-stranded β-barrel in the outer membrane. In TAAs, each monomer in the obligate trimer contributes four β-strands to the final barrel [10], but the nature of the β-barrel remains the same.

### Translocation via the Sec machinery

All type V secretion proteins pass the inner membrane via the Sec machinery. They are recognized by an N-terminal signal peptide, which is cleaved after translocation [10]. For most autotransporters, the signal peptide consists of a stretch of 20–30 amino acids that has a basic N-terminal region, a hydrophobic core, and a somewhat polar C-terminus but is highly variable in sequence. TAAs contain an N-terminal extension to the typical signal peptide [11]. This extension has implications in the transport process across the periplasmic space as it inhibits signal recognition particle (SRP) binding, slowing down the transport across the inner membrane [12]. This kinetic constraint may help to recruit periplasmic chaperones and the inner-membrane anchored protein TamB of the translocation and assembly module. We speculate that this prevents premature folding of TAAs in the periplasm and subsequent degradation of these proteins by periplasmic proteases.

### Folding constraints during outer membrane translocation

Outer membrane translocation of TAAs is initiated by the formation of a hairpin structure originating at the C-terminus of the passenger domain [13], which is also the area of highest sequence conservation among TAAs. The energy of translocation has to come from the folding process of the autotransporter itself, as the periplasm is devoid of ATP and no pH gradient exists at the outer membrane which could provide the energy for this process. This implies a mechanism in which the translocation is coupled to the export process at both membranes, similar to the Lpt pathway that transports lipopolysaccharides across both membranes [14]. As the C-terminal end of the passenger domain emerges from the β-barrel first, free energy can be obtained from “pulling out” the rest of the passenger domain from the barrel pore in a sequential manner [15]. This progressive folding model prevents retrograde slipping into the periplasmic space but also depends on additional factors to translocate passenger domains with intrinsically disordered domains. To obtain a complete model of translocation for TAAs, TAM and BAM must be considered as well as the Sec translocon and the periplasmic chaperones.

Translocation competence of autotransporter can be affected by the folding state of passenger domains in the periplasm. This was shown by experiments on chimeric passenger domains of the type Va cholera toxin B subunit fused with the *N. gonorrhoeae* IgA protease β-barrel domain in which the passenger domain could only be secreted when reducing agents were present which would disrupt disulphide bridges that would otherwise hinder translocation [16]. In TAAs, the A354P mutation in the C-terminal region of YadA affected translocation and folding competence even though the β-barrel domain was still inserted into the outer membrane [17]. These two examples show that even small changes to the autotransporter molecule can affect the translocation efficiency significantly. Further studies have led to the following general rules for translocation of passenger domains: (1) the sequence of the C-terminal region of the passenger domain is very important [18], (2) the net charge of the passenger domain influences the periplasmic folding state [19], and (3) secretion kinetics and translocation efficiency are correlated [20]. These findings need to be considered when creating chimeric proteins for surface display as recently shown by the creation of an affibody library fused to the translocator domain of the autotransporter Adhesin Involved in Diffuse Adherence (AIDA-I). Here, the authors exchange the passenger domain of the autotransporter AIDA-I from *Escherichia coli* with the “affibody” library system; affibodies are small (< 10 kDa), inert antibody mimetics each with a unique antigen binding site. This can potentially complement methods like phage display in the search for high affinity binding modules [21].

### Insertion and folding of TAAs into the outer membrane of gram-negative pathogens via BAM

BamA is crucial for the correct insertion of most OMPs with deletion of the gene leading to a lethal phenotype [22]. This is further highlighted by the fact that the BAM complex is responsible for the insertion of all transmembrane pore domains of type V secretion systems [8] with BamA as the central component in autotransporter biogenesis for trimeric autotransporters [23]. In the case of TAAs, trimerization of the β-barrel seems to occur in the periplasm [24]. This is consistent with the presence of trimeric periplasmic helper proteins like the inner membrane lipoprotein SadB, which is required for the TAA SadA to be displayed on the surface of *Salmonella typhimurium* [25] or the trimeric chaperone Skp of *E. coli* which forms 1:1 complexes with OMPs [26]. An extension of the BAM models discussed so far is needed to accommodate a full understanding of TAA insertion into the outer membrane.

Studies on the classical autotransporter NalP show that the pore of NalP could never be wide enough to accommodate a polypeptide segment with a tertiary structure [27]. By contrast, a three-hairpin intermediate was identified by NMR in the pore of a TAA [28]. Therefore, a barrel structure must exist with a large enough lumen to accommodate a partially folded polypeptide. All evidence points towards BamA to explain the contradictions.

### Role of TAM in bridging the gap between inner and outer membrane translocation

The energy needed for the export of proteins in gram-negative bacteria usually comes from the use of cytosolic ATP, the coupling of an electrochemical gradient at the inner membrane with the translocation process, or the entanglement of energy-dependent with energy-independent secretion steps to facilitate protein export [29]. Outer-membrane translocation on the other hand requires a more complex explanation due to the lack of ATP in the periplasm and the lack of an electrochemical gradient at the outer membrane [30]. A possible solution to the energy problem of the outer membrane translocation process would be a protein complex that spans the periplasmic space, thereby acting as a conduit between an energy-dependent transport occurring at the inner membrane and the energy-neutral process at the outer membrane. The TAM complex, which has been recently linked to the translocation process of (trimeric) autotransporters, could fill the gap for a unified model of autotransporter export [6].

**Fig. 1:**
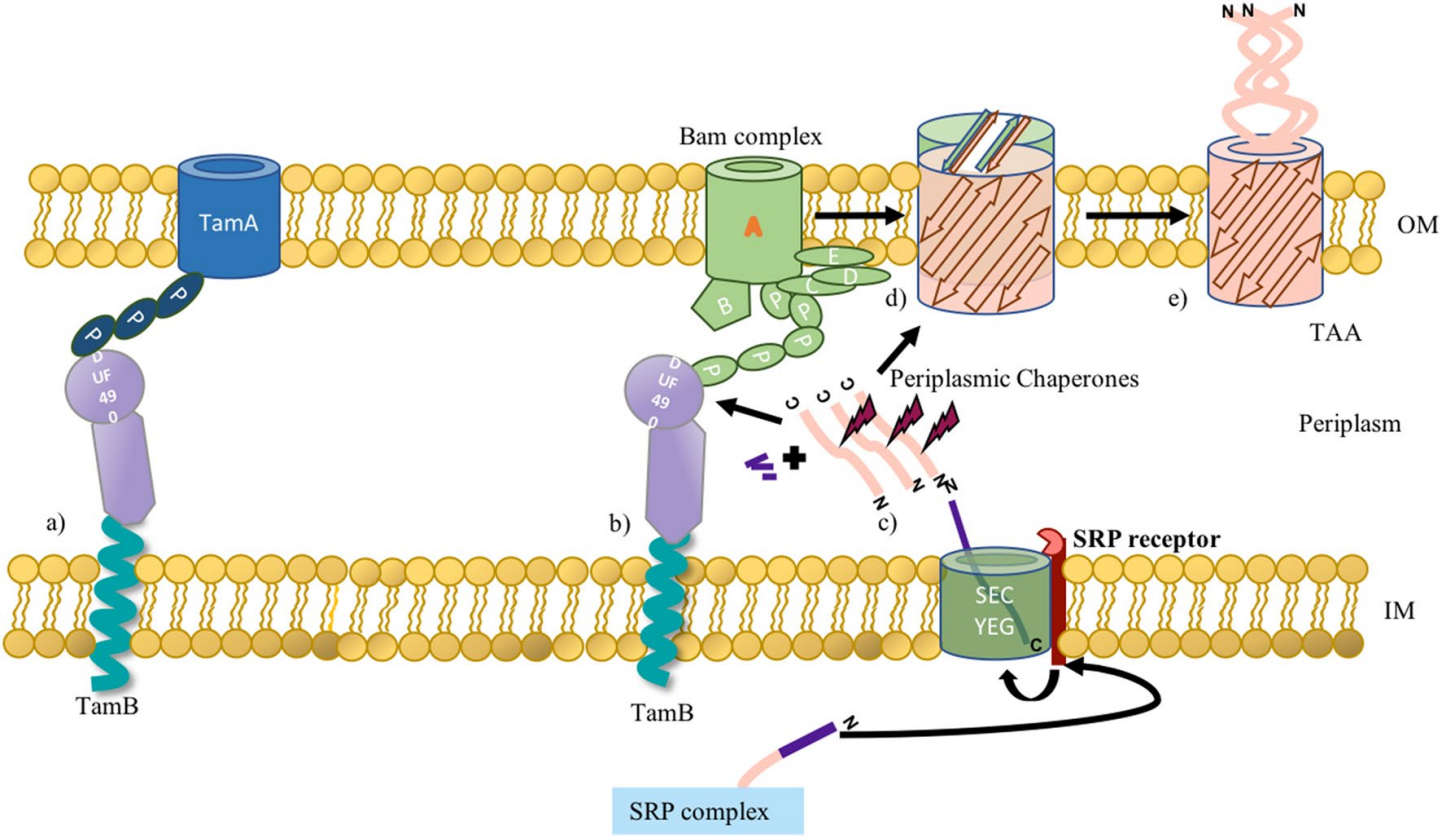
Schematic overview of translocation of TAAs through Inner membrane (IM) and Outer membrane (OM) of Gram-negative bacteria. **a** DUF 490 domain of TamB interacts with one of the POTRA (POlypeptide TRansport Associated) domains of TamA. **b** In the absence of TamA, TamB interacts with one of the POTRA domains associated with BamA. **c** TAAs transport through the IM into the periplasm as monomers, where periplasmic chaperons act to maintain the unfolded state of TAA monomers and TamB guides them to form a complex with BamA in OM. **d** Three TAA monomers together form a TAA: BamA complex. This complex allows TAA to form a twelve stranded β barrel (Anchor domain) to form a pore in the OM. **e** Completely exposed TAA on the surface of Gram-negative bacterium

The TAM complex consists of a 60 kDa outer membrane protein (OMP) of the Omp85 family, called TamA, which interacts with a conserved C-terminal domain of the inner-membrane anchored protein TamB via one of its three POlypeptide-TRansport-Associated (POTRA) domains [31]. These domains are protein–protein interaction modules with chaperone-like activity, the most prominent example being the five POTRA repeats in BamA that are essential for docking and folding of OMPs before insertion by the BAM [32]. TamA and TamB, together with the BAM, form a hetero-oligomeric translocation machinery that can accept autotransporters as a substrate [33]. This expands the so-far accepted model of outer membrane TAA translocation, which only depends on BAM, by a possible alternative pathway, in which TAM and BAM act in a concerted manner (Fig. 1).

TamA is very similar to BamA, with the major difference being the number of POTRA domains in the two proteins. This similarity may explain why in some species *tamA* is missing and at the same time *tamB* is residing on the same operon as *bamA,* with interactions of TamB and BamA confirmed in *Borrelia burgdorferi* [34]. In contrast to *tamA*, *tamB* homologs are present in most, if not all gram-negative pathogens [35]. This shows that, of the two TAM proteins, TamB is more important as it appears that the role of TamA in the translocation process can be performed by BamA [6].

### Head domains in TAAs

TAAs can be classified into repeats of head, connector, and stalk domains either as “lollipop structures” like YadA or as “beads-on-a-string” like BadA. They vary dramatically in size with YadA (422 aa) being one of the shorter TAAs with a length of about 23 nm [36], while BadA (3082 aa) is one of the longest TAAs, with a length of about 240 nm [37]. This initial classification originated from the assumption that the head domain, rich in β-strands, is the sole effector of protein function. It was thought that the stalk domain, formed from an α-helical coiled coil extended beyond the lipopolysaccharide (LPS) layer of gram-negative pathogens to allow the head domain to interact with various host factors [38]. While the head domains do mediate many molecular interactions ranging from autoagglutination to the attachment to extracellular matrix (ECM) components, the stalk domains also contribute; for instance the stalk of BadA binds to fibronectin [39] and two different regions of the stalk of EibD bind to IgA and IgG, respectively [40]. Therefore, to identify the interaction partners of a TAA, all regions of the passenger domain, not just the N-terminal head domains, must be considered.

**Fig. 2:**
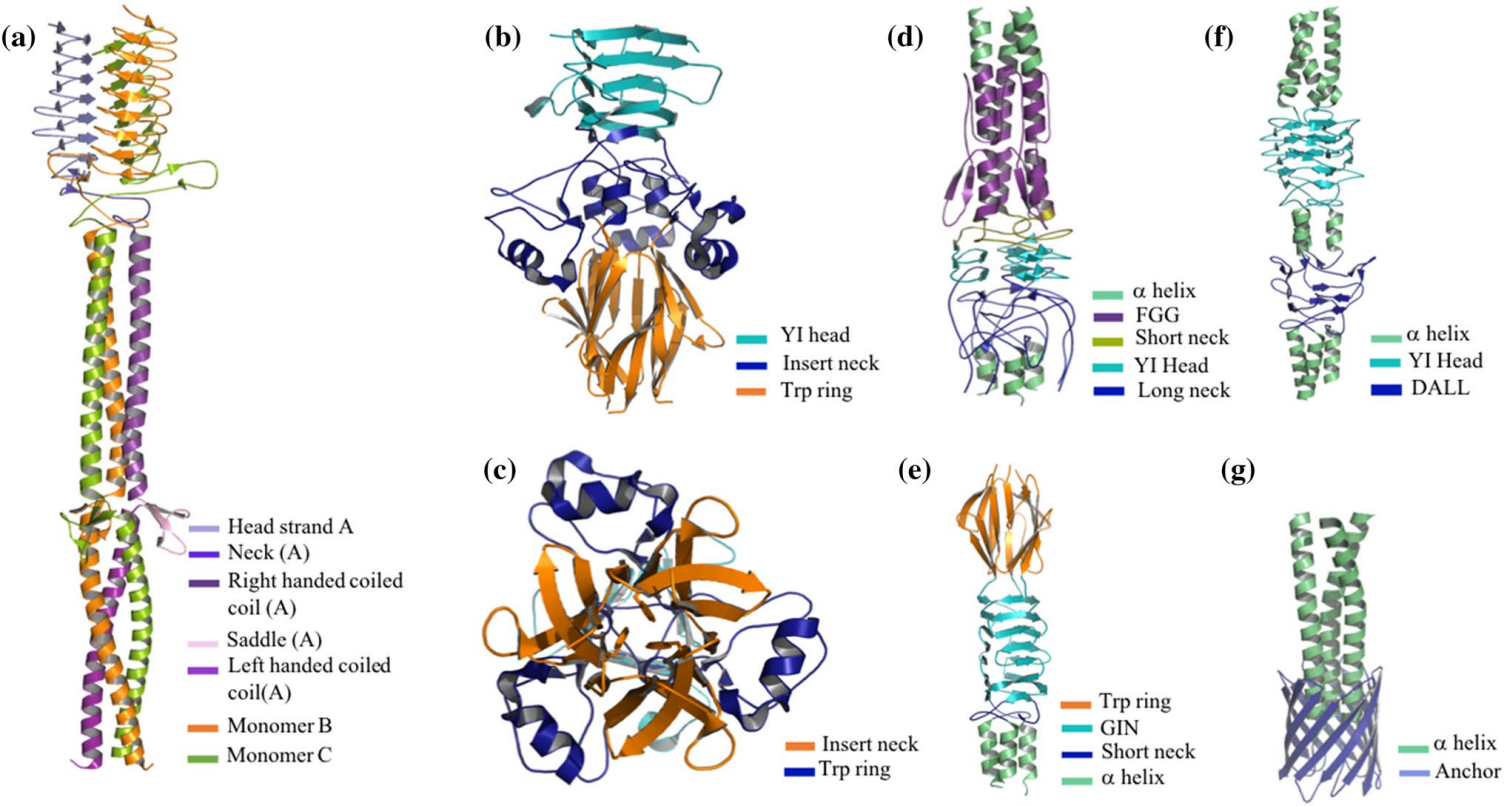
Different domains from TAA family of proteins. **a** The structure of EibD8 (PDB ID: 2XQH) is constituted from three identical intertwined monomers. The first monomer is coloured in different shades of purple for the head, neck, right-handed coiled coil, saddle and left-handed coiled coil domains of EibD, whereas the other two monomer chains are coloured in orange and green. **b** The crystal structure of binding domain 1 (BD1) of Hia (PDB ID:1S7M) divided into head (Teal), insert neck (Blue) and Tryptophan (Trp) ring (Orange) domains. **c** Top view of Hia BD1(PDB ID:1S7M) representing tryptophan side chain from each monomer. **d** Structure of BpaA (PDB ID:3LAA) representing FGG (Violet purple), short neck (Olive), head (Teal), long neck (Blue) domains. **e** Structure of BadA (PDB ID: 3D9X) representing Trp ring (Orange), GIN (Teal), short neck (Blue) domains. **f** Structure of SadA (PDB ID:2YO3) representing DALL (Blue) domain. **g** The structure of anchor domain of Hia (PDB ID:2GR7)

Head domains consist mainly of β-strands and can be divided into two types of structures. A very common motif is the so-called β-roll, as seen in the immunoglobulin-binding fragment of the TAA from *Escherichia coli* EibD (Head, Fig. 2a), in which two β-strands from each subunit contribute to a single superhelical turn, creating a left-handed solenoid-like structure [41]. The other type of structure is the β-prism module with each wall of the trimer being composed of a set of five β-strands. This structural element is present for example in the head domain of *Bartonella henselae* autotransporter BadA (GIN, Fig. 2e) [42]. Both of these motifs suffice to classify most of the TAA head domains, which is important to determine structural conservation inside each class.

### Structural conservation of TAA domains and motifs

The only part of a TAA that can be shown to be conserved based on sequence alone is the C-terminal anchor domain, consisting of the β-barrel domain and part of the anchoring coiled-coil in the barrel [11]. However, parts of passenger domains of one TAA often have high structural similarity to parts of another TAA, even if the similarity cannot be seen based on the sequence. One example are the left-handed β-roll “head domains” of Hia and BadA: they have superimposable folds but sequence identity of just 20% [42]. The structures cannot diverge as they have to trimerize in their native state, and the trimers are not formed by independently folded-domains, but by highly intertwined β-sheets and coiled-coil stalk domains [43]. This leads to the presence of short, structure-driven sequence patterns. Lupas and colleagues developed the bioinformatic tool daTAA [44], which treats domains of TAAs like a dictionary: short motifs or features are identified and compared to structures of representative exemplars for each domain. Conserved motifs allow the identification of structural elements by just “reading” the sequence of the passenger domain without any a priori information about the structure of the protein itself. This domain conservation can also be extended to other proteins, like the TrpRing and GIN domain of Hia and BadA which show yet again superimposable folds [31]. The number of common motifs for head and neck domains has been expanded, with motifs being split up in fine-grained submotifs as shown for the various neck domains (Table 1).

**Table 1:** List of domains from TAA family of proteins, their PDB ID, resolution, method with which the structure is solved and name and host organism of the protein

Name	Description	PDB ID	Resolution	Method	Organism
Anchor	Trimeric, 12-stranded β-barrel, responsible for pore formation in outer membrane of bacteria	2lme	–	Solid State NMR	*Yersinia enterocolitica serotype* 0:8
		2gr7	2.3 Å	X-Ray crystallography	*Haemophilus influenzae*
Stalk	Primarily right handed or left handed coiled-coil structure	2xqh	2.0 Å	X-Ray crystallography	*Enterobacteria phage P*
		3h7x	2.0 Å	X-Ray crystallography	*Yersinia enterocolitica*
FGG	Insertion of a 3-stranded antiparallel β strands into a stalk domain	2yo2	2.0 Å	X-Ray crystallography	*Salmonella typhimurium*
Saddle	Non-helical insertion which joins right handed coiled-coil region of stalk to left handed coiled-coil region in Eib protein	2xqh	2.0 Å	X-Ray crystallography	*Enterobacteria phage P*
Short neck	19 residues long β to α connector	3d9x	1.1 Å	X-Ray crystallography	*Bartonella henselae*
Long neck	22 residues long β to α connector	3laa	1.4 Å	X-Ray crystallography	*Burkholderia pseudomallei*
Insert necks	A neck variant with an extended insertion	1s7m	2.1 Å	X-Ray crystallography	*Haemophilus influenzae*
KG	Deletion variant of neck lacking the first β-strand	3emi	1.8 Å	X-Ray crystallography	*Haemophilus influenzae*
DALL	α to β connector	2ynz	1.4 Å	X-Ray crystallography	*Salmonella typhimurium*
		2yo3	2.0 Å	X-Ray crystallography	*Salmonella typhimurium*
HANS	Short variant α to β connector	2yo3	2.0 Å	X-Ray crystallography	*Salmonella typhimurium,*
Ylheads	Primarily β secondary structure, transverse in nature, perpendicular to the trimer axis. Most common form of head domain	1p9h	1.6 Å	X-Ray crystallography	*Yersinia enterocolitica*

**Fig. 3:**
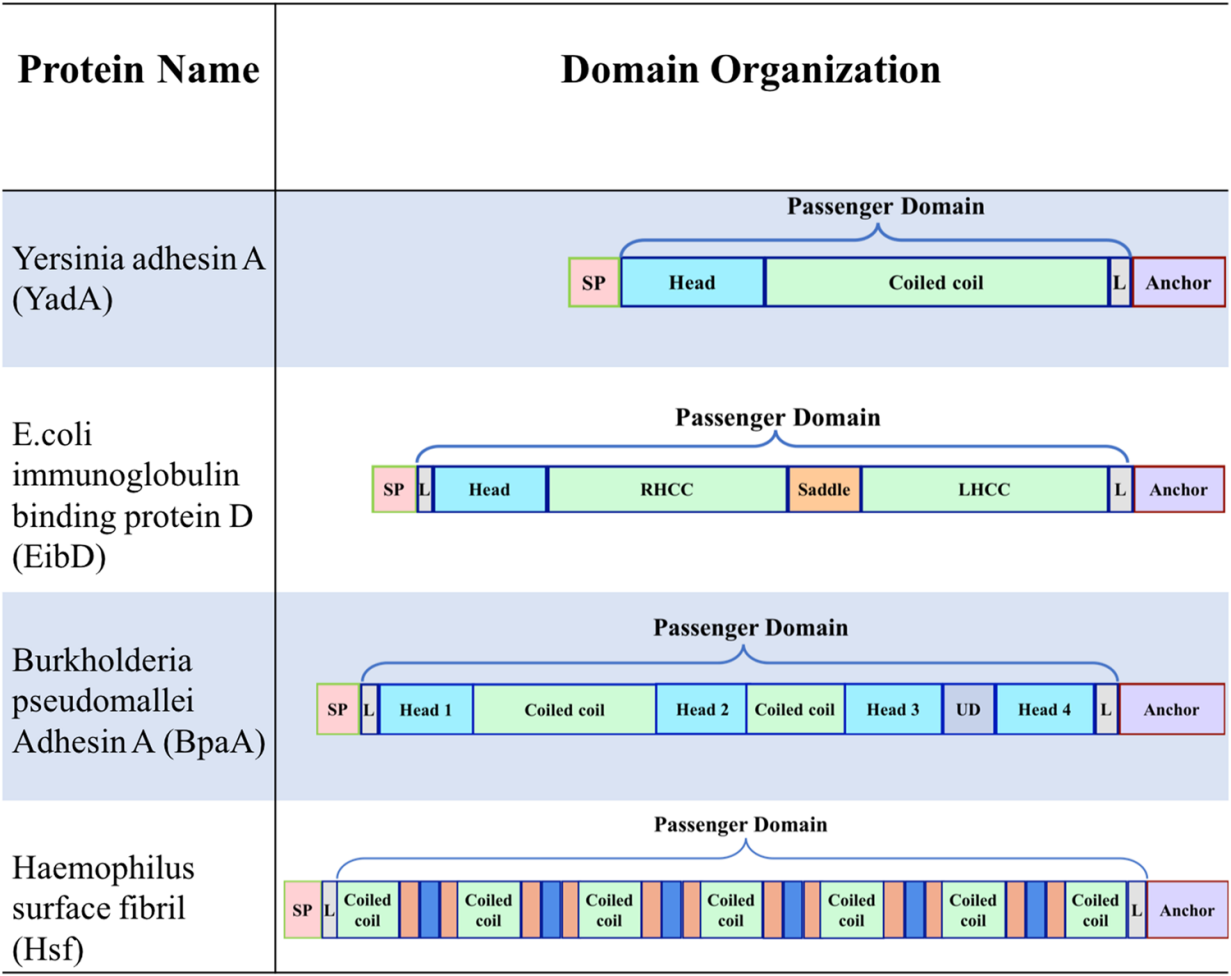
Domain organization in TAAs. Difference in domain organization of YadA, EibD, BpaA and Hsf. All TAAs contain a signal peptide (SP), passenger domain and anchor domain. The sub-domain organization in passenger domain varies among the proteins of TAA family. Head (cyan): YadA like heads, ‘L’ (grey): small linker regions; Coiled coil, RHCC (right-handed coil coil), LHCC (left-handed coiled coil) (green): coiled-coil stalk regions that vary in length and handedness. UD (grey): undefined domain. The Trp ring domains are another example of head and are shown by orange color, where as the blue color represents various neck domains present in Hsf

This allows the assembly of composite models using nearly invariant structures of connector domains such as necks and coiled-coil segments (Fig. 2) [45]. The order of the domains can vary from the classical head-neck-stalk motif and can be used to subcategorise different TAAs even further (Fig. 3). Structures of uncharacterized proteins can, therefore, be solved by a *divide*-*and*-*conquer* approach with individual fragments of head, connector, and stalk domains being combined to generate a complete model. For instance, the structure of the trimeric autotransporter SadA, one of the biggest TAA (1461 residues long, 148 kDa per monomer and about 108 nm as a trimer), has been modelled exactly this way [46].

Transferring this knowledge to yet uncharacterized proteins allows the identification of domain boundaries for expression studies and the use of molecular replacement for structure solution. For example, the structurally uncharacterized trimeric autotransporter BpaC from *Burkholderia pseudomallei* contains a TrpRing motif, followed by a GIN domain and a neck motif that initiates a coiled-coil segment at its N-terminus just as in BadA (3d9x, [42]). Furthermore, FGG motifs occur in BpaC as they do in BpaA (3la9, [47]), where they introduce a 120° rotation of the two linked coiled-coil segments. The identification of coiled-coils is made simple by the occurrence of neck motifs with the (DAVNxxQL) consensus, which in the published TAA structures signify the start of a coiled-coil segment as in BpaB (4usx, [48]). This allows a precise definition of the start of the coiled-coil heptad register (a)–(g), which is important when determining domain boundaries for use in the “divide-and-conquer” approach described above.

### Role of autotransporter expression in the infection process of prokaryotes

Autotransporters constitute the largest known family of virulence factors expressed by gram-negative bacteria and play prominent roles in invasion [49], serum resistance [50], adherence [51], biofilm formation [52], and many other pathogenic functions. Their primary function is the adherence to host cells which is the defining step in host invasion [53]. The increase in serum resistance by autotransporter expression allows the long-term survival of bacterial populations [54]. The role of TAAs in conferring serum resistance is mainly due to inference with complement activation as shown by YadA interaction with C4 binding protein (C4BP), accelerating the decay of C3 convertase and proteolytic cleavage of C4b, which ultimately inhibits the formation of the membrane attack complex (MAC) preventing lysis of the bacterium [55]. Hovingh and colleagues [55] have a comprehensive summary of bacterial proteins, including some TAAs that are known to evade the complement system.

### Trimeric autotransporters identified in *B. pseudomallei* play a significant role in different stages of the host infection process

*Burkholderia pseudomallei* is a motile gram-negative pathogen that lives in dead organic matter in tropical and sub-tropical soils and water [56]. It causes melioidosis, which is a febrile illness with acute and chronic disease states with a high mortality rate. *B. pseudomallei* can infect humans via the cutaneous or aerosol route upon contact with infected horses [57]. A major focus of research is on the prevention of infection by *B. pseudomallei*, as it is considered a potential bioweapon due to its ability to readily form infectious aerosols and the very low infectious dose required [58, 59], there has been an increase in research efforts, leading to successful vaccination in 2018 of mice using a live attenuated strain of *B. pseudomallei* expressing the trimeric autotransporter protein BatA, protecting them from a harmful aerosol infection [60]. Consequently, autotransporters are important in the virulence of this pathogen, implying that they may be targets for vaccine development against other gram-negative bacteria in the future. Although the majority of autotransporters in *B. pseudomallei* play some role in the virulence of the pathogen, only the TAA BpaC seems not only to increase virulence, but also to protect against macrophages and enhance serum survival [56]. Lafontaine et al. [61] showed in a series of adherence assays with human respiratory epithelial cells that deletion or overexpression of *bpaC* has different effects on the adhesion properties of the pathogen depending on the strain in which it is expressed. This strain-specific selectivity for certain types of epithelial cells implies either that there are other adhesion molecules compensating for the effect of the *bpaC* knockout deletion in *B. pseudomallei* compared to *B. mallei* or that other factors like a difference in thickness and composition of the LPS layer are responsible for the protein’s behaviour [62]. There is clearly a need for elucidating the exact molecular mode of action of this protein. Thus, BpaC is of particular interest in fully understanding *B. pseudomallei* pathogenicity.

### Future perspectives

The involvement of BpaC in the pathogenicity of *B. pseudomallei* highlights the ways in which TAAs are involved in the infection processes of gram-negative bacteria. BpaC fulfils various functions that allow the bacterium to adhere to certain epithelial cells, to survive and to proliferate in the host environment. Each function can be encoded on a different domain (Fig. 2): we speculate that this modular design allows the bacterium—in evolutionarily short timescales—to adapt to changes in the environment or the host. The structural conservation of trimeric autotransporter domains allows one to cross-correlate functional studies between two seemingly unrelated TAAs which make them of particular interest for the drug discovery pipeline from the structural biology point of view. A promising approach has been the development of a vaccine containing a recombinant virus that expresses the Va autotransporter BatA from *B. pseudomallei* as an antigen vector [60]. Studies in mice using this approach cleared an otherwise lethal aerosol dose of *B. pseudomallei* and *B. mallei*. The method also can serve as a blueprint for vaccination trials against other autotransporter antigens like BpaC.

Indeed vaccination seems to be a promising approach to protect against bacterial infections in which TAAs play an essential role. One successful bacterial vaccine is the multicomponent vaccine 4CMenB, which protects against *Neisseria meningitidis* serogroup B. Components of the vaccine target specifically the interaction of the TAA Neisserial adhesin A3 (NadA3) with its binding partner, the human receptor LOX-1. Liguori et al. [63] were able to demonstrate the specificity of NadA3 for LOX-1 by solving the structure of the N-terminal head domain and parts of the stalk of NadA3 and comparing it with the closely related TAA NadA5. This enabled them to identify key residues that explain the specificity of NadA3 towards LOX-1 [63]. A combination of functional studies and structural techniques demonstrates the power of structural-based vaccine design in the field of TAAs.

Alternative approaches of vaccination that are independent of a priori structrual information of the TAA-binding partner interaction also have been shown to be successful [64]. Here, the authors aimed to protect patients with chronic obstructive pulmonary disease using the TAA “*U*biquitous *S*urface *P*rotein” A2 (UspA2) from *Moraxella catarrhalis* as part of the vaccination cocktail against non-typeable *M. catarrhalis* [64].

There are still no structures showing how trimeric autotransporters interact with their binding partners. The very recent structure by electron tomography of the interaction between collagen and the TAA EtaA of the periodontal pathogen *Aggregatibacter actinomycetemcomitans*, though it shows the collagen fibril crossing the TAA, is not at sufficient resolution to reveal any details of the interaction [65]. The rapid progress in recent years in direct electron detectors, and the resulting revolution in resolution for single-particle cryo-electron microscopy and cryo-electron tomography, gives hope that structures of TAAs with their ligands will soon become possible. This can lead to a revolution in understanding of their interactions and so, potentially, in drug and vaccine design.
